# Long-Term Outcome of Proximal Gastrectomy for Upper-Third Advanced Gastric and Siewert Type II Esophagogastric Junction Cancer Compared With Total Gastrectomy: A Propensity Score-Matched Analysis

**DOI:** 10.1245/s10434-024-15048-8

**Published:** 2024-02-19

**Authors:** Seungho Lee, Yoon Soo Chae, Won-Gun Yun, Jane Chungyoon Kim, Jae Kyun Park, Min Gyu Kim, Jeesun Kim, Yo-Seok Cho, Seong-Ho Kong, Do Joong Park, Hyuk-Joon Lee, Han-Kwang Yang

**Affiliations:** Department of Surgery and Cancer Research Institute, Seoul National University Hospital, Seoul National University College of Medicine, Seoul, Republic of Korea

**Keywords:** Proximal gastrectomy, Advanced gastric cancer, Esophagogastric junctional cancer, Total gastrectomy

## Abstract

**Background:**

This study aimed to investigate the oncologic long-term safety of proximal gastrectomy for upper-third advanced gastric cancer (AGC) and Siewert type II esophagogastric junction (EGJ) cancer.

**Methods:**

The study enrolled patients who underwent proximal gastrectomy (PG) or total gastrectomy (TG) with standard lymph node (LN) dissection for pathologically proven upper-third AGC and EGJ cancers between January 2007 and December 2018. Propensity score-matching with a 1:1 ratio was performed to reduce the influence of confounding variables such as age, sex, tumor size, T stage, N stage, and tumor-node-metastasis (TNM) stage. Kaplan-Meier survival analysis was performed to analyze oncologic outcome. The prognostic factors of recurrence-free survival (RFS) were analyzed using the Cox proportional hazard analysis.

**Results:**

Of the 713 enrolled patients in this study, 60 received PG and 653 received TG. Propensity score-matching yielded 60 patients for each group. The overall survival rates were 61.7 % in the PG group and 68.3 % in the TG group (*p* = 0.676). The RFS was 86.7 % in the PG group and 83.3 % in the TG group (*p* = 0.634). The PG group showed eight recurrences (1 anastomosis site, 1 paraaortic LN, 1 liver, 1 spleen, 1 lung, 1 splenic hilar LN, and 2 remnant stomachs). In the multivariate analysis, the operation method was not identified as a prognostic factor of tumor recurrence.

**Conclusion:**

The patients who underwent PG had a long-term oncologic outcome similar to that for the patients who underwent TG for upper-third AGC and EGJ cancer.

**Supplementary Information:**

The online version contains supplementary material available at 10.1245/s10434-024-15048-8.

Gastric cancer is one of the most prevalent cancers and the fourth most common cause of cancer-related deaths worldwide.^1^ In eastern Asia, the incidence of gastric cancer is higher than in Western countries, as are disease mortality and morbidity.^2,3^ Recently, cardia/fundus gastric cancers, which are more frequent in Western countries, have been increasing in Korea.^4^ Therefore, research into understanding and treating upper gastric cancer has increased.

According to *Japanese Gastric Cancer Treatment Guidelines 2018* (5th edition), total gastrectomy (TG) is the standard surgical method for clinically node-positive (cN+) and for T2 to T4a upper-third and esophago-gastric junctional (EGJ) gastric cancers.^5^ In addition, proximal gastrectomy (PG) can be applied exclusively to proximal cT1N0 gastric cancers or some of the early or advanced esophago-gastric junctional cancers smaller than 4 cm in size where more than half of the distal stomach can be preserved.^5^

Proximal gastrectomy has several advantages over TG in terms of body weight maintenance, postoperative anemia, and nutritional aspects including vitamin B12, protein, albumin, and cholesterol.^6–9^ In addition, a meta-analysis found that laparoscopic PG showed operative benefits such as a shorter operating time and less blood loss.^10^

Despite the aforementioned advantages, PG has rarely been performed for advanced gastric cancers (AGCs) due to omitted dissection of distal lymph nodes (LNs), including numbers 4d, 5, 6, and 12, and because oncologic safety has always been a primary concern when PG is performed for AGC patients. To determine the feasibility of PG for AGC, we previously reported oncologic factors related to distal LN metastasis in upper-third and EGJ gastric cancer.^11^ We found that pathologic T2 stage and pathologic T3 with less than 5 cm of tumor showed no metastasis in distal LNs regardless of middle-third invasion. This study retrospectively analyzed the feasibility of PG for AGC patients and distal LN metastasis incidence compared with TG patients using the propensity score-matching (PSM) method.

## Method

### Patients and Study Design

Between January 2007 and December 2018, 1237 patients with AGC at the upper-third level of the stomach or EGJ cancer underwent curative PG or TG with standard LN dissection and no neoadjuvant chemotherapy at the Seoul National University Hospital. Our study was approved by the Institutional Review Board of Seoul National University Hospital (H-2009-095-1157).

From the 1237 patients, the study excluded those who had a double lesion of gastric cancer (*n* = 1) and those who had received palliative operation (*n* = 86) or a combined resection, which indicated that the patients had received other organ resection, such as hepatectomy, adrenalecromt, salphingo-oophorecromy, or the like (*n* = 480). We retrospectively reviewed the prospectively collected electronic medical records of operative and postoperative outcomes, recurrence, and survival.

The patients were divided into PG and TG groups according to the type of surgery performed. The primary end points were disease-free survival, cancer-specific survival, and overall survival, and the secondary end points were pathologic and operative outcome, postoperative complication, and recurrence pattern. For LN dissection, D1+ LN dissection included stations 1, 2, 3a, 4sa, 4sb, 7, 8, 9, and 11p, whereras D2 included LN stations removed in D1+ as well as stations 11d, and/or 10 in the PG group. In the TG group, D1 included stations 1, 2, 3, 4sa, 4sb, 4d, 5, 6, and 7, and D2 included D1 with stations 8a, 9, 11p, 11d, and 12a. Postoperative complications were classified according to the Clavien-Dindo system.^12^

### Propensity Score-Matching

Propensity scores were calculated per patient, with confounding factors related to the following oncologic factors: age, gender, tumor size, pathologic T staging, pathologic N staging, and pathologic tumor-node-metastasis (TNM) staging. Matching was performed one-on-one using the nearest-neighbor-matching method.

### Statistical Analysis

All statistical analyses were performed using the SciPy library of Python (version 1.10.0).^13^ Descriptive data are presented as mean ± standard deviation or median (range). The Kaplan-Meier method was used to analyze the survival data using the Lifelines Library of Python (version 0.27.4).^14^ Significance was defined by a *p* value lower than 0.05.

## Results

### Patient Characteristics

**Fig. 1:**
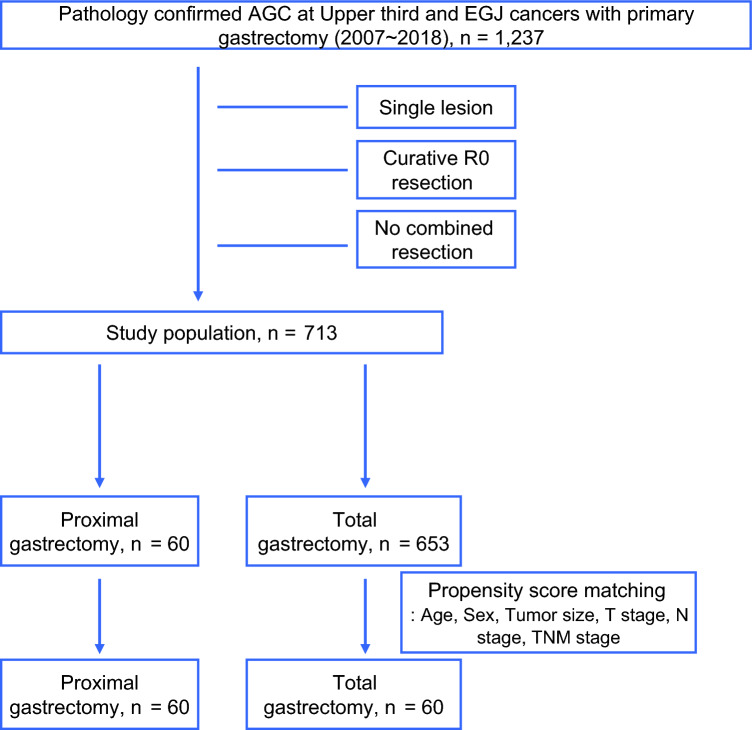
Study design.

Among the 713 patients enrolled in the final analysis, 60 had PG and 653 had TG (Fig. 1). After PSM using age, sex, tumor size, T stage, N stage, and TNM stage, the standardized mean difference across the covariates decreased (Fig. S1).

Before PSM, the clinical characteristics of the two groups differed significantly (Table 1). The patients in the TG group showed a significantly higher fraction of pathologic T4 and N3 tumor than those in the PG group (34.6 % vs 10.0 %, *p* < 0.001; 8.3 % vs 30 %, *p* < 0.001, respectively, chi-square test). In addition, only one patient (1.7 %) with American Joint Committee on Cancer (AJCC) seventh edition stage IIIc disease appeared in the PG group (TG: 125 [19.1 %], *p* <0.001, chi-square test). Not only was the pathologic stage higher in the TG group, but tumor size also was significantly greater (5.0 vs 3.25 cm, *p* < 0.001, *t* test).

**Table 1:** Patients and pathologic characteristics

Variables		Whole cohort			After PSM		
		PG (*n* = 60) *n* (%)	TG (*n* = 653)* n* (%)	*p* value	PG (*n* = 60) *n* (%)	TG (*n* = 60) *n* (%)	*p* Value
Mean age (years)		62.2 ± 12.3	59.6 ± 11.6	0.100	62.2 ± 12.3	61.8 ± 11.0	0.839
Gender	Male	41 (68.3)	459 (70.3)	0.865	41 (68.3)	43 (71.7)	0.842
	Female	19 (31.7)	194 (29.7)		19 (31.7)	17 (28.3)	
Approach	Open	42 (70.0)	534 (81.8)	0.033	42 (70.0)	42 (70.0)	0.105
	Laparoscopic	18 (30.0)	111 (17.0)		18 (30.0)	14 (23.3)	
	Robot	0 (0.0)	8 (1.2)		0 (0.0)	4 (6.7)	
Retrieved LNs: *n* (range)		33.0 (24.8–45.5)	48.0 (37.0–61.0)	<0.001	33.0 (24.8–45.5)	46.5 (34.8–60.5)	<0.001
Lauren	Intestinal	25 (43.9)	238 (37.3)	0.107	25 (43.9)	31 (53.4)	0.131
	Diffuse	20 (35.1)	319 (50.0)		20 (35.1)	22 (37.9)	
	Mixed	11 (19.3)	70 (11.0)		11 (19.3)	3 (5.2)	
	Unknown	1 (1.8)	11 (1.7)		1 (1.8)	2 (3.4)	
T stage	T2	34 (56.7)	168 (25.7)	<0.001	34 (56.7)	34 (56.7)	0.635
	T3	20 (33.3)	259 (39.7)		20 (33.3)	23 (38.3)	
	T4a	5 (8.3)	209 (32.0)		5 (8.3)	3 (5.0)	
	T4b	1 (1.7)	17 (2.6)		1 (1.7)	0 (0.0)	
N stage	N0	35 (58.3)	218 (33.4)	<0.001	35 (58.3)	38 (63.3)	0.612
	N1	13 (21.7)	118 (18.1)		13 (21.7)	13 (21.7)	
	N2	7 (11.7)	121 (18.5)		7 (11.7)	3 (5.0)	
	N3	5 (8.3)	196 (30.0)		5 (8.3)	6 (10.0)	
Stage	Ib	21 (35.0)	101 (15.5)	<0.001	21 (35.0)	25 (41.7)	0.664
	IIa	21 (35.0)	117 (17.9)		21 (35.0)	19 (31.7)	
	IIb	7 (11.7)	108 (16.5)		7 (11.7)	9 (15.0)	
	IIIa	4 (6.7)	99 (15.2)		4 (6.7)	1 (1.7)	
	IIIb	6 (10.0)	103 (15.8)		6 (10.0)	4 (6.7)	
	IIIc	1 (1.7)	125 (19.1)		1 (1.7)	2 (3.3)	
Median tumor size: cm (IQR)		3.25 (2.5–5.0)	5.0 (3.5–7.5)	<0.001	3.25 (2.5–5.0)	3.5 (2.6–5.3)	0.934
Median PRM: cm (IQR)		1.3 (0.8–2.2)	2.0 (1.2–3.5)	<0.001	1.3 (0.8–2.2)	2.2 (1.1–3.3)	0.003
LI	No	30 (50.0)	266 (40.9)	0.17	30 (50.0)	36 (60.0)	0.359
	Yes	30 (50.0)	384 (59.1)		30 (50.0)	24 (40.0)	
VI	No	56 (93.3)	489 (75.2)	<0.001	56 (93.3)	48 (80.0)	0.057
	Yes	4 (6.7)	161 (24.8)		4 (6.7)	12 (20.0)	
NI	No	42 (70.0)	257 (39.5)	<0.001	42 (70.0)	37 (61.7)	0.442
	Yes	18 (30.0)	393 (60.5)		18 (30.0)	23 (38.3)	

PSM, propensity score-matching; PG, proximal gastrectomy; TG, total gastrectomy; LN, lymph node; IQR, interquartile range; PRM, proximal resection margin ; LI, lymphatic invasion; VI,vascular invasion; NI, neural invasion

After PSM, the two groups showed similar results for most clinical characteristics including surgical approach (*p* = 0.105), T stage (*p* = 0.635), N stage (*p* = 0.612), TNM stage (*p* = 0.664), tumor size (*p* = 0.934), lymphatic invasion (*p* = 0.359), vascular invasion (*p* = 0.057), and neural invasion (*p* = 0.442) using chi-square for categorical analysis and *t* test for tumor size. However, the PG and TG patients differed significantly in number of retrieved LNs (*p* < 0.001, *t* test) and length of proximal resection margins (*p* = 0.003, *t* test), even after PSM.

### Surgical Outcome

Among the PSM-matched patients, a similar distribution of Siewert II and upper-third advanced gastric cancer was shown between the PG and TG groups (*p* = 0.128, Fisher’s exact test; Table 2). The TG patients had a significantly longer operation time than the PG patients (*p* = 0.002, *t* test). Most of the PG patients received esophagogastrostomy(*n* = 55) rather than double-tract reconstruction (*n* = 5). In addition, the same number of D1+ LN dissections was performed in both groups (*n* = 19). However, the results were similar for hospital stay (*p* = 0.474, *t* test) and early complications exceeding grade III Clavien-Dindo classification (*p* = 0.675, Fisher’s exact test). Detailed postoperative complications are described in Table 3. After operation, slightly more patients in the TG group received adjuvant chemotherapy, but the difference was not statistically significant (*p* = 0.143, Fisher’s exact test). After operation, the incidence of reflux gastritis was significantly higher in the PG group (*p* < 0.001, Fisher’s exact test).

**Table 2:** Surgical outcome

	PG (*n* = 60) *n* (%)	TG (*n* = 60) *n* (%)	*p* value
Tumor position			0.128
Siewert II	3 (5.0)	9 (15.0)	
Upper third	57 (95.0)	51 (85.0)	
Mean operation time (min)	191.4 ± 61.6	224.9 ± 51.4	0.002
Reconstruction			<0.001
Esophagojejunostomy	0	60 (100)	
Esophagogastrostomy	55 (90.0)	0	
Double tract	5 (10.0)	0	
Lymph node dissection			1.000
D1+	19 (0.31)	19 (0.31)	
D2	41 (0.69)	41 (0.69)	
Mean hospital stay (days)	10.4 ± 4.7	11.0 ± 5.5	0.474
Adjuvant CTx			0.143
None	36 (60.0)	27 (45.0)	
Yes	24 (60.0)	33 (55.0)	
Op-related early Cx (Clavien-Dindo ≥III)			0.675
None	58 (96.7)	56 (93.3)	
Yes	2 (3.3)	4 (6.7)	
Reflux gastritis			<0.001
None	41 (68.3)	57 (95.0)	
Yes	79 (31.7)	3 (5.0)	

PG, proximal gastrectomy; TG, total gastrectomy; Ctx, chemotherapy; Op, operation; Cx, complications

**Table 3:** Landscape of postoperative complicationd

Op-related early Cx	PG (*n* = 60) *n* (%)	TG (*n* = 60 *n* (%)
Grade 0	46 (76.7)	41 (68.3)
Grade 1		
Wound	1 (1.7)	0
Pulmonary	0	1 (1.7)
Undetermined	0	1 (1.7)
Grade 2		
Wound	3 (5.1)	1 (1.7)
Fluid collection	2 (3.4)	4 (6.8)
Motility disorder	1 (1.7)	0
Pulmonary	1 (1.7)	3 (5.1)
Other infection	1 (1.7)	1 (1.7)
Cardiac	0	1 (1.7)
Neuropsychic	1 (1.7)	0
Undetermined	2 (3.4)	3 (5.1)
Grade 3		
Fluid collection	1 (1.7)	0
Intraabdominal bleeding	0	1 (1.7)
Luminal bleeding	0	1 (1.7)
Pulmonary	0	1 (1.7)
Renal	0	1 (1.7)
Grade 4		
Undetermined	1 (1.7)	0

PG, proximal gastrectomy; TG, total gastrectomy

### Oncologic Outcome

**Fig. 2:**
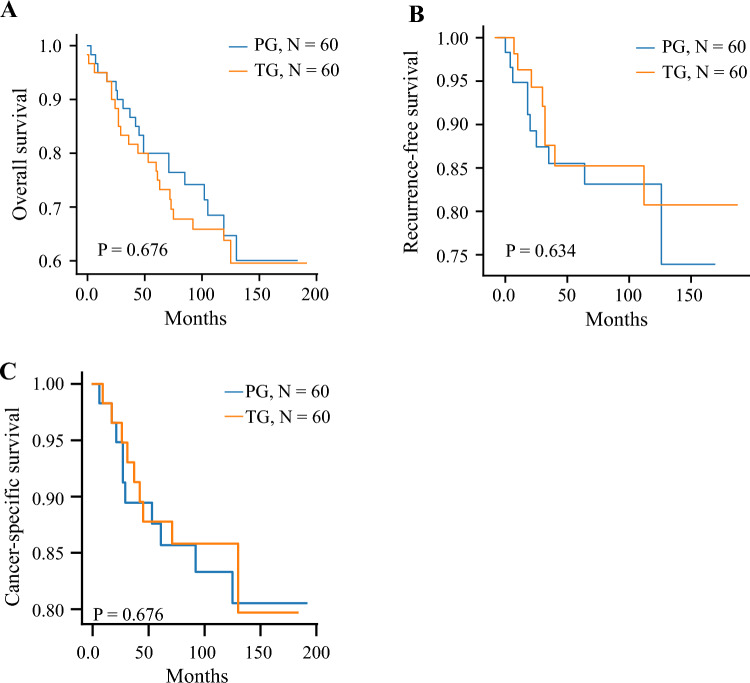
Kaplan-Meier survival analysis for the proximal gastrectomy and total gastrectomy groups. (**A**) Overall survival, (**B**) recurrence-free survival, and (**C**) cancer-specific survival for patients with upper third AGC or Siewert type II EGJ cancer who underwent proximal gastrectomy or total gastrectomy. The *p* value was calculated by the log-rank test. AGC, advanced gastric cancer; EGJ, esophagogastric junction cancer

We first comparatively analyzed long-term oncologic outcomes between the matched PG and TG groups (Fig. 2). The median follow-up period was 123 months for the PG group and 92 months for the TG group. Not only was overall survival similar, but recurrence-free survival also showed similar results during 10 years (*p* = 0.676 and *p* = 0.634, log-rank test, respectively; Fig. 2A and B). In addition, both groups showed similar survival rates for the patients who died due to gastric cancer progression (*p* = 0.676, log-rank test; Fig. 2C). Among the 60 PG patients, 8 recurrences were reported, and among the TG patients, 10 recurrences after gastrectomy were reported (Table 3). Although the landscapes of the recurrence sites were similar overall, two remnant stomach cancers were reported in the PG group and 5 peritoneal seedings were reported in the TG group. In addition, none of the cancers metastasized to distal LNs including LN stations 4d, 5, 6, and 12a. One case of station 10 LN metastases and para-aortic node metastasis was found among the PG patients.

Finally, we performed Cox regression analysis to calculate the hazard ratio of each feature (Table 4). In the univariate Cox regression analysis, well-known oncologic features, including AJCC stage, lymphatic invasion, vascular invasion, and neural invasion were associated with significantly shorter recurrence-free survival. However, operation method did not increase the rate of recurrence in the PSM cohort (hazard ratio [HR], 1.26; 95 % confidence interval [CI], 0.49–3.2; *p* = 0.633)

**Table 4:** Landscape of recurrence after gastrectomy

Recurrence Site	PG (*n* = 8) *n* (%)	TG (*n* = 10) *n* (%)	*p* value
Anastomosis,	1 (12.5)	0 (0.0)	0.188
Bone	0 (0.0)	1 (10.0)	
LN station 10	1 (12.5)	0 (0.0)	
Liver	1 (12.5)	2 (20.0)	
Lung	1 (12.5)	1 (10.0)	
Paraaortic LN	1 (12.5)	1 (10.0)	
Peritoneum	0 (0.0)	5 (50.0)	
Remnant stomach	2 (25.0)	0 (0.0)	
Spleen	1 (12.5)	0 (0.0)	

PG, proximal gastrectomy; TG, total gastrectomy; LN, lymph node

## Discussion

In this study, we analyzed long-term oncologic outcomes and recurrence patterns of pathologic AGC patients who received PG. In propensity score-matched patient data, we found similar overall survival and oncologic outcomes, including cancer-specific survival and disease-free survival, between PG and TG in pathologic confirmed AGC patients. In 120 matched patients, operation method was not identified as a prognostic marker, whereas oncologic factors, including AJCC stage, lymphatic invasion, vascular invasion, and neural invasion, were significant prognostic features of recurrence after both TG or PG.

After PG, distal LN metastasis and remnant stomach cancer are the biggest issue in terms of oncologic safety. Because risk for both problems are increasing if the tumor is in an advanced stage, PG for upper-third or EGJ advanced cancer usually is not recommended.^5^ Therefore, several studies focused only on distal LN metastasis and incidence of remnant gastric cancer after PG. Rosa et al.^15^ not only demonstrated similar overall survival between the PG and TG groups in both upper third of EGC and AGC, but also reported similar postoperative complication rates between the two groups. In 329 propensity score-matched AGC patients, Peng et al.^16^ also reported no significant difference between PG and TG, finding no number 5 or 6 LN metastasis in pathologic T2/3 or Borrmann types I and II tumors when size was less than 4 cm. Related to LN metastasis pattern, Sato et al.^17^ insisted that radical PG may be indicated for patients who have EGJ with gastric invasion length shorter than 40 mm due to the low risk of LN station 3b metastasis. In addition, a very low metastatic rate at LN stations 4d and 12a and no metastasis of LN station 5 or 6 were found among the 202 patients with T2/T3 AGC who had TG.^18^

We also identified risk factors associated with distal LN metastasis and found that pathologic T stage, tumor size, and invasion of the middle third were significant factors.^11^ Although 11 patients were at risk for distal LN metastasis according to our previous study (including 5 patients with T3 tumors larger than 5 cm and 6 patients with T4 tumors), no LN metastasis or recurrence at LN stations 4d, 5, 6, or 12a was observed in the PG group. This result may have been due to an insufficient number of patients to demonstrate distal LN metastasis. Therefore, in this study, even with the absence of distal LN metastasis, it could not be conclusively stated that PG was entirely safe for patients at risk for distal LN metastasis.

Regarding remnant stomach cancer, several studies reported a higher incidence of remnant stomach cancer after PG than after distal gastrectomy.^19^ In addition, only two patients in the PG group showed remnant stomach cancer, and one of these patients had EGC diagnosed during the regular endoscope follow-up exam and survived more than 10 years. Therefore, regular follow-up examination after PG is necessary to prevent late detection of remnant stomach cancer and improve cancer-specific survival.

In addition to distal LN metastasis and remnant stomach cancer, our data showed similar surgical and long-term oncologic outcomes. In contrast, several studies supported the finding that PG has several advantages over TG in terms of body weight maintenance, postoperative anemia, and nutritional aspects.^6–9^ Taken together, to evaluate the risk for recurrence of PG for AGC patients, not only are prospective randomized trials needed, but also large scale clinical data analysis. However, considering the nutritional benefit as well as the similar long-term survival and recurrence after gastrectomy, PG is a reasonable treatment for both advanced gastric cancer located in the upper third of the stomach and Siewert type II EGJ cancer. In addition, we believe that new surgical technology such as real-time imaging technology using near-infrared fluorescence (NIF) with indocyanine green (ICG) may be helpful in determining the surgical approach due to its capability of detecting metastatic nodes with a false-negative rate of 1 %.^20,21^

## Conclusion

In summary, in terms of oncologic safety, patients with upper-third AGC or Siewert type II EGJ cancer may be considered as candidates for PG with standard LN dissection.

### Supplementary Information

Below is the link to the electronic supplementary material.

Supplementary Figure 1. Propensity score matching result. A. Barplot showed substantially decreased standardized mean differences after matching. (PPTX 70 kb)
