# Supplementary figures and images for: Long-Term Outcome of Proximal Gastrectomy for Upper-Third Advanced Gastric and Siewert Type II Esophagogastric Junction Cancer Compared With Total Gastrectomy: A Propensity Score-Matched Analysis
Source: Ann Surg Oncol. 2024 Feb 19;31(5):3024–30. doi: 10.1245/s10434-024-15048-8 (PMC10997683; doi:10.1245/s10434-024-15048-8)

## Slide 1
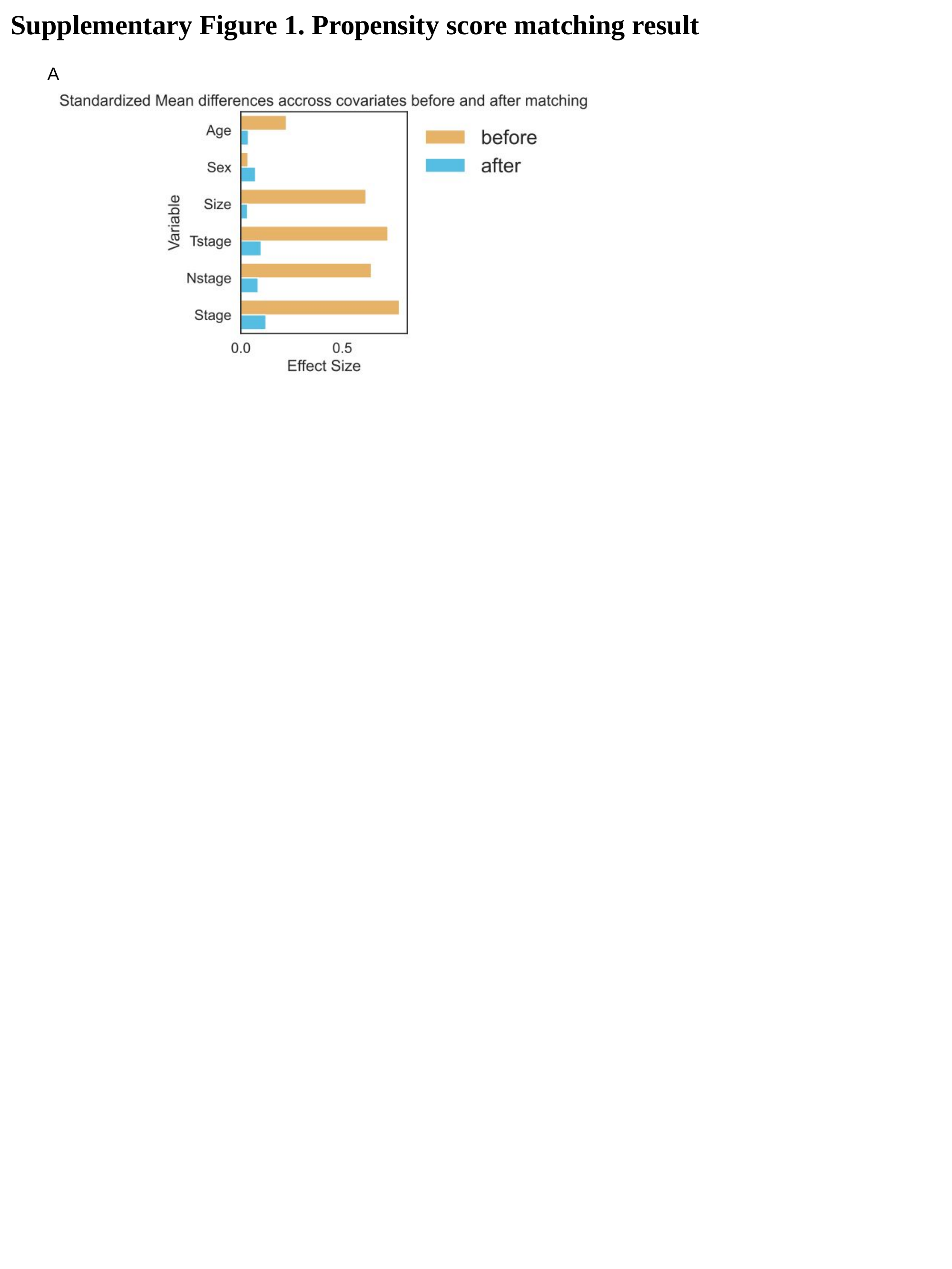

Supplementary Figure 1. Propensity score matching result
A

Supplement: Supplementary file 1 — Supplementary Figure 1. Propensity score matching result. A. Barplot showed substantially decreased standardized mean differences after matching. (PPTX 70 kb) [file 10434_2024_15048_MOESM1_ESM.pptx]
